# Traumatic Bilateral Luxatio Erecta from a Sliding Injury Down a Ladder; A Rare Case Report and Literature Review 

**DOI:** 10.29252/beat-070216

**Published:** 2019-04

**Authors:** Saptarshi Biswas, Ronald Peirish

**Affiliations:** 1 *Attending Surgeon, Forbes Hospital, Allegheny Health Network, Pennsylvania, USA*; 2 *Medical Student, Lake Erie College of Osteopathic Medicine, Erie, Pennsylvania, USA*

**Keywords:** Bilateral luxatio erecta, Bilateral inferior shoulder dislocations, Trauma

## Abstract

Bilateral inferior shoulder dislocations also known as luxatio erecta is an extremely rare injury that is commonly complicated with injuries to the humeral head, glenoid, clavicle, scapula, rotator cuff, capsule, ligaments, brachial plexus, axillary artery and vein.  Our patient is a 66-year-old man who presented with both upper extremities above his head in a fixed abducted position after sliding down a ladder approximately 6-meters. Initial radiographs revealed both humeral heads to be located below the glenoid fossa with each humeral shaft parallel to the scapular spines.  Computed tomography (CT) revealed a right Hill-Sachs compression fracture (posterolateral humeral head) with a bony Bankart fracture (anteroinferior glenoid) and an avulsion fracture of the left acromion. Successful closed reduction was obtained.  Upon follow up, bilateral rotator cuff tears were suspected and confirmed with magnetic resonance imaging (MRI).  Early recognition, treatment and follow-up is essential to minimize complications.

## Introduction

Shoulders are the most commonly dislocated joint due to its dependence on the soft tissue. The shoulder is minimally constrained by bone. There are three types of shoulder dislocations; anterior, posterior and inferior [1-4]. Bilateral shoulder dislocations are rare with posterior being the most common [5]. The most common causes of shoulder dislocations are due to electrical shock, extreme trauma and epilepsy [6]. Bilateral luxatio erecta is an extremely rare condition with 29 cases identified in the literature [7]. 

Skeletal, soft-tissue, neurological and vascular injuries are commonly encountered with this injury.  80% of patients will have a fracture of the greater tuberosity or tear to the rotator cuff [8]. Fractures of the acromion, clavicle, coracoid, greater tuberosity and humeral head can be seen as well.  Luxatio erecta can result from two different mechanisms-direct and indirect, with indirect being more common [9, 10]. Prompt recognition and treatment is required by physicians to minimize complications. Patients with this injury have a unique presentation both radiographically and clinically.  Radiographically, the humeral head will be below the glenoid with the humeral shaft parallel to the spine of the scapula and the affected arm in a fixed abducted position with the elbow flexed and the hand behind the head [7,11,12]. In this paper, we discuss a rare case of traumatic bilateral luxatio erecta with bilateral rotator cuff tears and fractures of the humeral head, glenoid and acromion.  

## Case Report

**Fig. 1:**
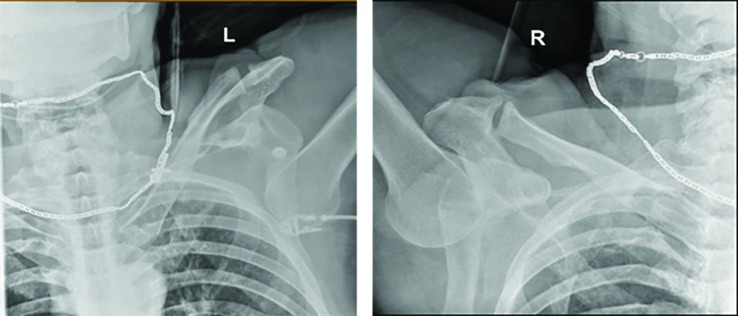
Bilateral shoulder radiography taken on admission which demonstrates bilateral luxatio erecta

A 66-year-old man presented to our emergency department (ED) after sliding down a ladder approximately 6-meters after the ladder kicked out from underneath him. When emergency medical services (EMS) arrived on scene they witnessed a man lying in the prone position with his face on a pile of wood and arms raised neck high.  The left shoulder had obvious deformity with subjective loss of sensation.  The right shoulder appeared normal with intact sensation. In the emergency room, the patient underwent a primary and secondary survey in accordance with the Advanced Trauma Life Support (ATLS) protocol.  Physical examination revealed both shoulders to be above his head in a fixed position with extreme pain with any attempted shoulder movement.  Radiographs (Figure 1) confirmed the suspected diagnosis of bilateral inferior shoulder dislocations.

**Fig. 2:**
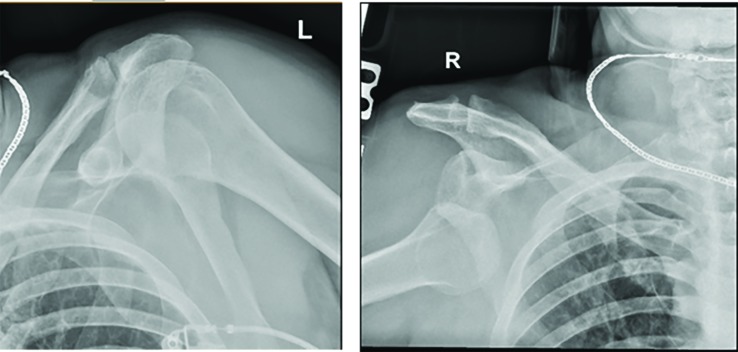
Bilateral shoulder radiography taken after initial attempted reduction, which demonstrates interval reduction of the left shoulder (Left) and right shoulder luxatio erecta (Right)

**Fig. 3:**
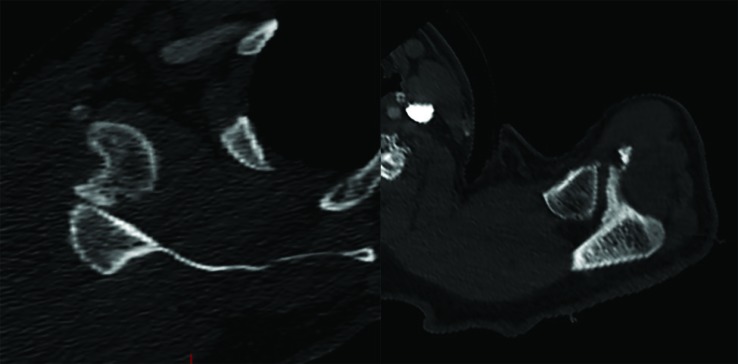
Computerized tomography (CT) scan reconstruction images post-initial reduction. Hill-Sachs Impaction and Bankart fracture seen on the right shoulder (Left image). Avulsion fracture of left acromion (Right image)

Closed reduction under anesthesia (Fetanyl 50mcg, Etomidate 5mg, Midazolam 2mg and Ketamine 40mg administered intravenously) was performed by the emergency room physician with external rotation and axial traction to the left shoulder.  The right shoulder was unable to be reduced after multiple attempts.  Post reduction radiographs (Figure 2) were obtained which demonstrated interval reduction of the left shoulder and unsuccessful reduction of the right shoulder.  Shoulder 3-D CT reconstruction images (Figure 3) were obtained which revealed the right shoulder to be dislocated anterioinferiorly with an acute Hill-Sachs impaction fracture and an acute Bankart fracture measuring 5mm. The left shoulder was dislocated anteriorly with an avulsion fracture of the lateral acromion. The patient reported decreasing pain in the left shoulder and continued pain in the right shoulder.  Orthopedic surgery was consulted for further management.

**Fig. 4:**
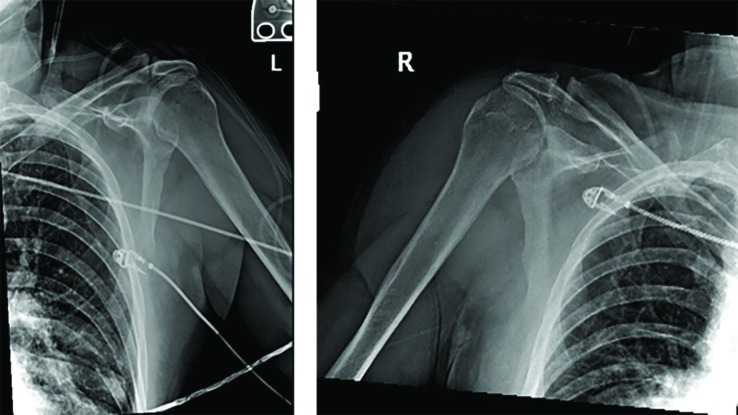
Radiography taken after second attempted reduction, which demonstrates successful reduction

**Fig. 5:**
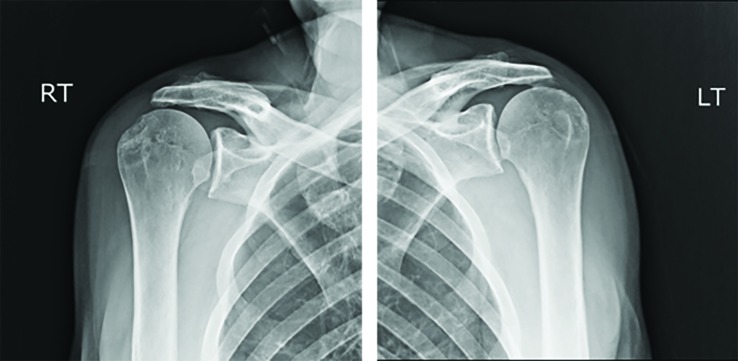
Radiography taken 2-weeks after discharge, which demonstrates bilateral high-riding humeral heads with concentrically reduced shoulder joints

Closed reduction was performed by the orthopedic surgeon with pain medication (Fentanyl 100mcg administered intravenously). The right shoulder was reduced with traction-countertraction and the left shoulder was reduced with traction with anterior and downward pressure.  Bilateral reductions were felt to be achieved after a palpable clunk and the ability of a smooth, pain free shoulder range of motion.  Post-reduction radiographs (Figure 4) confirmed successful reduction.  Bilateral well-padded shoulder slings were placed.  The patient was instructed to be non-weight bearing and limit shoulder motion for 2-3 weeks.  He was admitted for observation and discharged two days later. Two weeks following discharge, the patient presented to the orthopedic clinic.  Radiographs (Figure 5) were obtained which demonstrated bilateral high-riding humeral heads with reduced shoulder joints.  On physical exam, the patient was unable to actively flex either shoulder.  An MRI was ordered on both shoulders which revealed the left shoulder to have a massive full thickness tear of the supraspinatus and infraspinatus retracted to the level of the glenoid.  In addition, the subscapularis was torn, and the long head of the biceps dislocated medially in the intertubercular groove. The right shoulder revealed a full thickness tear of the supraspinatus and infraspinatus with 3-3.5cm of retraction. Two months after his initial injury, the patient underwent a left shoulder arthroscopic extensive glenohumeral joint debridement. Pre-operatively the operating surgeon planned to perform a superior capsular reconstruction but abandoned this as surgery revealed extensive cartilage loss throughout the shoulder joint. Based on the intra-operative findings, arthroplasty was recommended at a later time. 

## Discussion

The shoulder joint is complex in that it has four separate articulations (sternoclavicular, glenohumeral, acromioclavicular and scapulothoracic joint) which allows for the largest range of motion of any joint in the body.  The glenoid fossa is shallow comprising of only one-quarter to one-third the surface of the humeral head.  Stability is derived from the capsuloligamentous structures and the musculotendinous cuff [13]. Luxatio erecta can result from two mechanisms- direct and indirect.  The direct mechanism, less common is caused by an axial compression through a fully abducted arm.  This causes the humeral head to perforate through the inferior glenohumeral ligament. The indirect mechanism, more common is when an abducted arm is struck with a hyperabducted force, which causes the proximal humerus to be levered out of the acromion injuring the inferior, middle glenohumeral ligaments and rotator cuff [1,9,10]. Our patient experienced an indirect mechanism of injury.

Our patient’s presentation was classic radiographically and clinically which allowed for prompt recognition and understanding of the importance of early shoulder reduction. Adequate sedation and analgesia is necessary to successfully reduce this injury. There are two reduction techniques that can be used. The traction-countertraction technique is performed with superiorly directed traction with gradual adduction to the shoulder with an assistant providing counter-traction by a sheet wrapped around the upper torso.  In the two-step technique, lateral pressure is applied to the humeral shaft with one hand and the other hand pulls superiorly on the medial epicondyle. These forces cause the humeral head to rotate anteriorly and superior allowing for the conversion into an anterior shoulder dislocation. Once this conversion is achieved any anterior dislocation reduction technique can be utilized [1, 14]. Our patient’s attempted reductions highlight the importance of understanding each reduction technique.

Our patient experienced several additional injuries, which included bilateral rotator cuff tears, Hill-Sachs compression fracture with a bony Bankart fracture on the right and an avulsion fracture of the left lateral acromion.  Mallon* et al.* reported that about 80% of luxatio erecta have fractures of the greater tuberosity or rotator cuff tears [8].  It has beenreported that approximately 50% of inferior shoulder dislocations have injuries to the rotator cuff [15].  In our case, the orthogonal radiographs obtained in office demonstrated bilateral high-riding humeral heads indicative of large rotator cuff tears.  Untreated large rotator cuff injuries can cause chronic pain, decrease range of motion and rotator cuff arthropathy, so surgical intervention would need to be considered. Mallon* et al* concluded that 37% of inferior shoulder dislocations have an associated fracture [8]. Nambiar* et al.* reported that proximal humerus fractures occurred in 39% of cases with 4% of these being Hill-Sachs compression fractures. In addition, they found that scapula fractures occurred in 8% of cases with 7% being glenoid and 0.5% acromion [7]. A structured rehabilitation program consisting of three phases is paramount following a shoulder dislocation.  Phase I focuses on using a shoulder sling for comfort and gentle passive shoulder exercises to prevent stiffness and reduce pain.  Phase II works to improve range of motion and muscle strength.  Special attention will be placed on the rotator cuff, trapezius and serratus anterior muscles.  Isometric muscle contractions, flexibility and theraband exercises will be used during this phase.  Phase III attempts to regain shoulder control and proprioception.  During this phase, resistance exercises will be used [16].

In conclusion, luxatio erecta is a well-recognized type of shoulder dislocation but is still considered a rare injury especially when bilateral.  A thorough history and physical examination is essential.  Orthogonal radiographs are required to confirm that the humeral head is below the rim of the glenoid and the humeral shaft parallel to the scapular spine.  Additional imaging (CT and MRI) can aid in diagnosing associated injuries.  An understanding of the reduction techniques (traction-countertraction and two-step) is needed to prevent additional injury to the shoulder.  Prompt orthopedic consultation should be used if difficulty arises with initial reduction attempts.  Even if initial reduction is successful, orthopedic consultation and follow up is highly recommended due to associated injuries.    

## References

[1] Youm T, Takemoto R, Park BK (2014). Acute management of shoulder dislocations. J Am Acad Orthop Surg.

[2] Karaoglu S, Guney A, Ozturk M, Kekec Z (2003). Bilateral luxatio erecta humeri. Arch Orthop Trauma Surg.

[3] Laskin RS, Sedlin ED (1971). Luxatio erecta in infancy. Clin Orthop Relat Res.

[4] Brady WJ, Knuth CJ, Pirrallo RG (1995). Bilateral inferior glenohumeral dislocation: luxatio erecta, an unusual presentation of a rare disorder. J Emerg Med.

[5] Meena S, Saini P, Singh V, Kumar R, Trikha V (2013). Bilateral anterior shoulder dislocation. J Nat Sci Biol Med.

[6] Rezazadeh S, Vosoughi AR (2011). Closed reduction of bilateral posterior shoulder dislocation with medium impression defect of the humeral head: a case report and review of its treatment. Case Rep Med.

[7] Nambiar M, Owen D, Moore P, Carr A, Thomas M (2018). Traumatic inferior shoulder dislocation: a review of management and outcome. Eur J Trauma Emerg Surg.

[8] Mallon WJ, Bassett FH 3rd, Goldner RD (1990). Luxatio erecta: the inferior glenohumeral dislocation. J Orthop Trauma.

[9] Davids JR, Talbott RD (1990). Luxatio erecta humeri A case report. Clin Orthop Relat Res.

[10] Pandey V, Madi S, Tapashetti S, Acharya K (2015). Rotator cuff tears in luxatio erecta: an arthroscopic perspective of two cases. BMJ Case Rep.

[11] Kothari K, Bernstein RM, Griffiths HJ, Standertskjöld-Nordenstam CG, Choi PK (1984). Luxatio erecta. Skeletal Radiol.

[12] Freundlich BD (1983). Luxatio erecta. J Trauma.

[13] Culham E, Peat M (1993). Functional anatomy of the shoulder complex. J Orthop Sports Phys Ther.

[14] Brophy RH, Marx RG (2009). The treatment of traumatic anterior instability of the shoulder: nonoperative and surgical treatment. Arthroscopy.

[15] Padgham M, Walker JS (1996). Inferior glenohumeral dislocation (luxatio erecta humeri). J Am Osteopath Assoc.

[16] Vines A (2013). Rehabilitation after shoulder dislocation: information for patients.

